# Supplementary material for: Maternal Exposure to Ozone and the Risk of Birth Defects: A Time-Stratified Case-Crossover Study in Southwestern China
Source: Toxics. 2024 Jul 19;12(7):519. doi: 10.3390/toxics12070519 (PMC11281228; doi:10.3390/toxics12070519)
Supplement: Supplementary file 1 [file toxics-12-00519-s001.zip › Table S1.pdf]

Table S1. The results of the Z-tested after subgroup analyses.

			**Preconception**			**First-trimester**		
			**3rd**	**2nd**	**1st**	**1st**	**2nd**	**3rd**
**Age**								
≥35	vs	<35	1.760	1.219	-0.142	-0.213	-1.321	-1.913
**Ethnicity**								
Han	vs	Other	0.296	-0.390	-0.336	0.000	0.152	-0.546
**Conception**								
＞2	vs	≤2	1.473	0.563	0.441	-0.330	-0.787	-1.617
**Fertility**								
≥ 2	vs	＜2	-0.567	0.225	0.000	0.441	0.229	0.000
**Education**								
Low	vs	Middle	0.523	1.676	1.580	1.439	1.019	0.369
Middle	vs	High	-0.958	-0.568	0.380	1.398	**1.999**	1.529
Low	vs	High	0.259	-1.439	**-2.164**	**-2.976**	**-2.976**	-1.820
**Incoming**								
High	vs	Middle	0.893	-1.302	**-2.061**	**-2.950**	**-3.430**	**-2.713**
Middle	vs	Low	0.748	0.103	0.069	-0.440	-0.861	-0.746
High	vs	Low	-1.232	0.484	0.854	1.845	**2.478**	**2.074**
**Domicile**								
Urban	vs	Rural	1.305	0.553	-0.092	-1.390	**-2.230**	-1.759
**Sex**								
Boy	vs	Girl	-1.173	-1.613	-1.696	-0.565	0.346	0.799
