# Supplementary figures and images for: Maternal Exposure to Ozone and the Risk of Birth Defects: A Time-Stratified Case-Crossover Study in Southwestern China
Source: Toxics. 2024 Jul 19;12(7):519. doi: 10.3390/toxics12070519 (PMC11281228; doi:10.3390/toxics12070519)

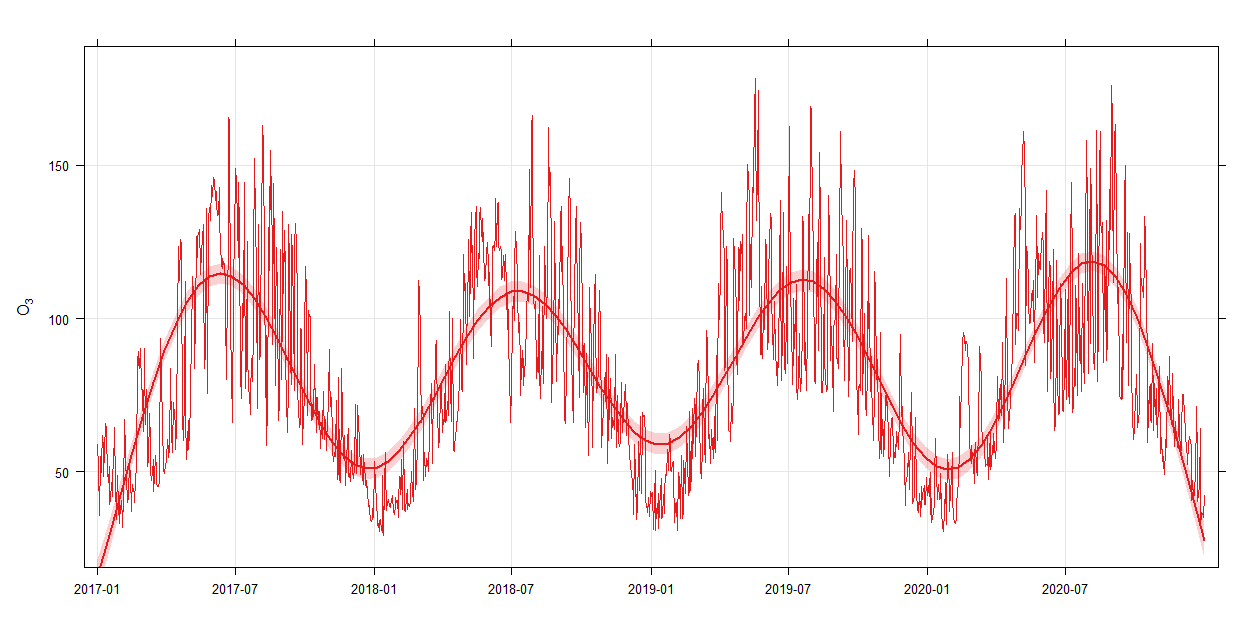

Supplement: Supplementary file 1 [file toxics-12-00519-s001.zip › Figure S1.tiff]
